# Optic nerve sheath diameter is associated with outcome in severe Covid-19

**DOI:** 10.1038/s41598-022-21311-3

**Published:** 2022-10-14

**Authors:** Jakob Pansell, Peter C. Rudberg, Max Bell, Ola Friman, Charith Cooray

**Affiliations:** 1 The Department of Clinical Neuroscience, Karolinska Institutet, Stockholm, Sweden; 2 The Department of Anesthesia and Intensive Care Medicine, Karolinska University Hospital, Stockholm, Sweden; 3 The Department of Physiology and Pharmacology, Karolinska Institutet, Stockholm, Sweden; 4 The Department of Clinical Neurophysiology, Karolinska University Hospital, Stockholm, Sweden

**Keywords:** Medical research, Neurology, Viral infection

## Abstract

Neurological symptoms are common in Covid-19 and cerebral edema has been shown post-mortem. The mechanism behind this is unclear. Elevated intracranial pressure (ICP) has not been extensively studied in Covid-19. ICP can be estimated noninvasively with measurements of the optic nerve sheath diameter (ONSD). We performed a cohort study with ONSD ultrasound measurements in severe cases of Covid-19 at an intensive care unit (ICU). We measured ONSD with ultrasound in adults with severe Covid-19 in the ICU at Karolinska University Hospital in Sweden. Patients were classified as either having normal or elevated ONSD. We compared ICU length of stay (ICU-LOS) and 90 day mortality between the groups. 54 patients were included. 11 of these (20.4%) had elevated ONSD. Patients with elevated ONSD had 12 days longer ICU-LOS (95% CI 2 to 23 p = 0.03) and a risk ratio of 2.3 for ICU-LOS ≥ 30 days. There were no significant differences in baseline data or 90 day mortality between the groups. Elevated ONSD is common in severe Covid-19 and is associated with adverse outcome. This may be caused by elevated ICP. This is a clinically important finding that needs to be considered when deciding upon various treatment strategies.

## Introduction

Neurological symptoms and complications are common in Covid-19. High levels of biomarkers of neuronal damage have been recorded^1,2^. In an early post-mortem series of patients that had died with Covid-19 there was evidence of neuroinflammation in many patients and mild to moderate cerebral edema in nearly half of all cases^3^. The pathophysiologic mechanisms are not clear but one known factor is the increased risk of thromboembolism and micro thrombotic events in Covid-19. Other suggested mechanisms include hyperinflammation resulting in neural or vascular complications, autoimmune disorders and encephalopathy. Persistent hypoxemia and multiple organ failure also occur in severe Covid-19 and may result in hypoxic neuronal injuries^1,2^. Elevated intracranial pressure (ICP) is a known cause of secondary brain injury in other pathologies^4^ but has not been extensively studied in severe Covid-19. A few studies of invasively measured ICP in Covid-19 have been performed in less severe cases. In one such study elevated ICP was diagnosed upon lumbar puncture in a number of patients with Covid-related headache^5^. Three studies using noninvasive estimation of ICP in severe Covid-19 have been published^6–8^. One of these studies suggest that elevated estimated ICP could be associated with a longer Intensive Care Unit Length of Stay (ICU-LOS) but not with short-term mortality in severe Covid-19^6^.

Studies of the possible mechanism of neurological symptoms and complications in Covid-19 are few and small and no long-term follow-up has to our knowledge been done. It is well-known that ICP can be estimated by measuring the Optic Nerve Sheath Diameter (ONSD) with sonography^9,10^. The optic nerve sheath contains a subarachnoid space with circulating cerebrospinal fluid (CSF). When ICP increases, a volume shift occurs, leading to more CSF circulating in the subarachnoid space surrounding the optic nerve. This dilates the optic nerve sheath and increases ONSD. Limitations to ONSD as an ICP estimate are e.g. individual baseline variations of ONSD and inter-rater reliability^10^. ONSD correlates with eye diameter (ED)^11^. Adjusting for ED can lead to better precision when estimating ICP with ONSD, by partly mitigating for individual baseline variations. ONSD divided by ED increased precision in ICP estimation in two previously published studies^12,13^ and in one study from our research group, currently undergoing peer review. We therefore used this approach, with a method that has been described in previous publications^14^. In the two previously published studies the optimal cut-off to identify elevated ICP was 0.26^13^ and 0.25^12^ respectively. The study from our research group, currently undergoing review, was larger than both of these studies and used the same protocol as we used in this study. The optimal cut-off in that study was 0.295. We therefore set this as the threshold for elevated ONSD/ED. A recent study from our group showed that ONSD measurements could be performed with excellent inter-rater reliability using a standardized protocol^14^.

The aim of this study was to explore ONSD in patients with severe covid-19 and examine it for possible associations with ICU-LOS and 90 day mortality.

## Methods

### Ethical considerations

The study was conducted in accordance with the Helsinki declaration and was approved by the Swedish Ethical Review Authority, record number 2020-03004. The requirement for informed consent from the study subjects was waived by the Swedish Ethical Review Authority due to the nature of the cohort that makes informed consent unfeasible. ONSD ultrasound is a safe, noninvasive, and painless procedure that can be performed without interfering with patient care. The cut-off for elevated estimated ICP was unknown to ONSD operators during the data collection. ICP estimation therefore could not influence clinical decisions or treatment strategies. We informed the patients’ next of kin and gave them right to opt out on behalf of the patient.

### Patient cohort

#### Inclusion criteria

All patients ≥ 18 years old treated for Covid-19 in the ICU at Karolinska University Hospital in Stockholm during the time-period November 2020 to April 2021, sedated or unconscious and on invasive ventilation, were eligible for inclusion. Exclusion criteria were ocular disease or ocular trauma. We used a convenience sample since our two ONSD operators had a heavy clinical workload in the ICU during this period. Eligible patients were included if available for ONSD examination when an ONSD operator was available. We were therefore not able to measure all patients at the same time during their ICU stay.

### Clinical data collection

Measurements were performed by two experienced ultrasound operators. Both had theoretical and practical training in ONSD ultrasound with at least 30 exams prior to this study. We used a protocol we developed based on the CLOSED protocol^15^. Our operators have shown excellent inter-rater reliability when using this protocol^14^. We performed ONSD ultrasound with a General Electrics GE Vivid S70 machine using a linear 11L-probe. Power was reduced to achieve a Mechanical Index < 0.23 and frequency was kept at 10 MHz as outlined in the CLOSED protocol for ONSD sonography^15^. ONSD and ED were both averaged from measurements in the transversal and the sagittal plane for each eye. Color Doppler was utilized to visualize the central retinal artery and/or vein to properly identify the optic nerve and its direction. ONSD was measured perpendicular to the optic nerve, three millimeters behind the retina.

We recorded baseline data including age, sex, comorbidities, the ICU day for ONSD/ED measurement, ratio of pO2/FiO2 (PFI), pCO2, the occurrence of acute kidney injury (AKI) and the need for vasopressor and/or inotropic support. We retrospectively added data on PEEP, pressure support/pressure control setting, ventilator mode, accumulated hours of prone positioning before ONSD exam, as well as ICU-LOS, 90 day mortality and number of days alive during the first 90 days from admission to the ICU, from electronical patient charts.

### Exposure and outcomes

We corrected for individual variations of ONSD baseline by dividing ONSD with ED, as previously suggested^12,13^. We set a cut off for exposure of elevated ONSD/ED at ≥ 0.295 mm. Outcome measures, comparing patients with and without high ONSD/ED, were ICU-LOS and mortality within 90 days from ICU admission.

### Statistical analysis

Patients were divided into two groups: elevated ONSD/ED (≥ 0.295 mm) and normal ONSD/ED (< 0.295 mm). ICU-LOS in both groups was tested for normality with the Shapiro–Wilk’s test and subsequently compared with 95% CI, using a two-sample t-test with unequal variances and significance level set at 0.05. The outcomes 90 day mortality and ICU-LOS dichotomized at ≥ 30 days were compared between groups using Fisher’s exact test. A Kaplan–Meier survival graph with 95% CI was produced for survival during the first 90 days since ICU admission. Continuous baseline data in both groups was tested for normality using the Shapiro Wilk’s test and subsequently compared with 95% CI, using a two-sample t-test with unequal variances and significance level set at 0.05. We used Fisher’s exact test to compare binary baseline data between groups. Median day of ICP estimation was compared between the two groups using Fisher’s exact non-parametric equality of medians test. We performed a linear regression analysis on day of ICP estimation and ONSD/ED.

Sensitivity analyses for the potential effects of extreme values in this relatively small data set were performed by sequentially excluding patients from both groups with high ICU-LOS (> 50 days), low ICU-LOS (< 5 days) and patients with high or low ICU-LOS. We also performed a sensitivity analysis by removing patients who died in the ICU to avoid confounding of ICU-LOS by ICU mortality.

All calculations and graphs were performed and created in Stata, v 14.2.

### Ethics approval and consent to participate

This study adheres to The Helsinki declaration and was approved by the Swedish Ethical Review Authority, record number 2020-03004. The requirement for informed consent from the study subjects was waived by the Swedish Ethical Review Authority due to the nature of the cohort that makes informed consent unfeasible. Next of kin were informed and given the right to opt out on behalf of the patient.

## Results

We performed measurements in 55 patients from November 2020 to April 2021. One patient was excluded upon request from next of kin and 54 patients were included in the final analysis. There were no attempted measurements of ONSD that did not succeed. Measurements were performed once per patient. All patients were sedated at time of measurement. Median day for ONSD measurement after ICU admission was day six with an interquartile range of 4 to 13 (see Table 1 for baseline data of the cohort).

**Table 1:** Baseline data.

**Demographics**	
Age (mean/SD)	64/± 13
Male (n/%)	43/79.6%
**Comorbidities**	
Cardiovascular disease (n/%)	10/18.5%
Asthma/COPD (n/%)	6/11.1%
Diabetes (n/%)	14/25.9%
Obesity (n/%)	19/35.2%
Hypertension (n/%)	21/38.9%
Previous stroke (n/%)	3/5.6%
Traumatic brain injury (n/%)	1/1.9%
Hydrocephalus/chronic intracranial hypertension (n/%)	0/0%
Pregnancy (n/%)	1/1.9%
Known allergy to sonographic gel	0/0%
**Treatments**	
Vasopressors (n/%)	38/70.4%
Inotropic drugs (n/%)	6/11.1%
Ventilator mode, pressure support (n/%)	25/46.3%
Ventilator mode, pressure control (n/%)	29/53.7%
PEEP (mean/SD)	11.3/± 2.9
Pressure support/pressure control (mean/SD)	13.8/± 4.5
Peak airway pressure (mean/SD)	25/± 5
Accumulated time of prone positioning (hours/SD)	38/± 34
**Other baseline data**	
PFI (ratio of pO2 in kPa to FiO2) at time of ONSD/ED measurement (mean/SD)	19/± 6
pCO2 (kPa) at time of ONSD/ED measurement (mean/SD)	6.6/± 1.5
AKI at time of ONSD/ED measurement (n/%)	10/18.5%
ICU day of ONSD/ED measurement (median/interquartile range)	6/4 to 13

*SD* standard deviation, n numbers, *COPD* chronic obstructive pulmonary disease, *ONSD/ED* optic nerve sheath diameter divided by eyeball diameter, *PFI* ratio of partial pressure of oxygen (kPa) in arterial blood divided by fraction of inspired oxygen, *pCO2* partial pressure of carbon dioxide (kPa) in arterial blood, *AKI* acute kidney injury, *ICU* intensive care unit.

Loss to follow up on ICU-LOS occurred in one patient due to transfer to a hospital with a different system for electronic patient charts. In one patient there was no available data for pCO2 within a reasonable temporal proximity to the ICP estimation. None of these two patients had an elevated ONSD/ED.

**Figure 1:**
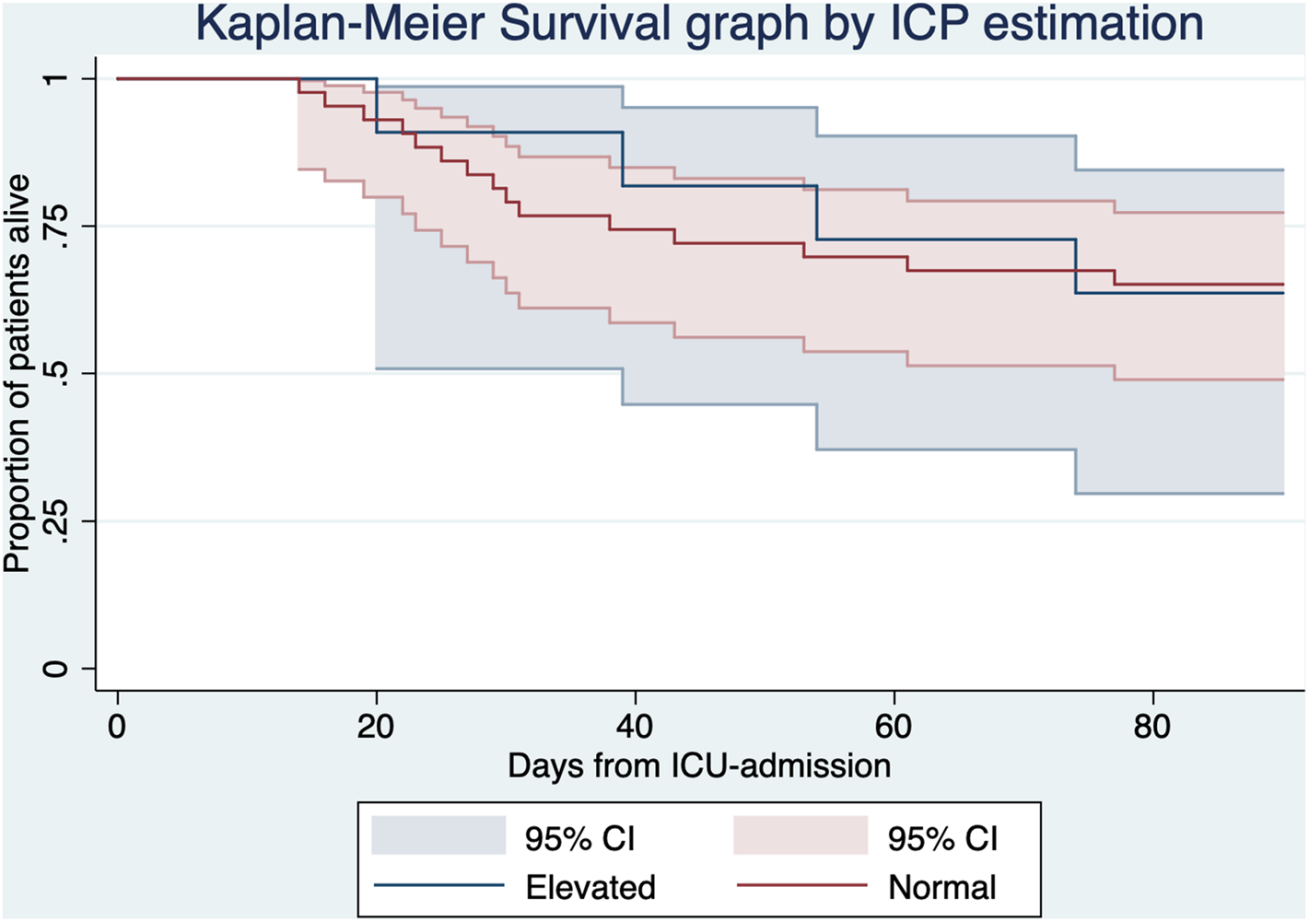
90 days survival.

11 out of 54 patients (20.4%) had an elevated ONSD/ED (≥ 0.295 mm). Patients with an elevated ONSD/ED had a mean ICU-LOS of 38 days (95% CI 26 to 50). Patients with a normal ONSD/ED had a mean ICU-LOS of 26 days (95% CI 21 to 30). The difference between the two groups of 12 days in ICU-LOS was significant (95% CI 2 to 23, p = 0.03). The risk ratio for long ICU-LOS (≥ 30 days) in ICU survivors with elevated ONSD/ED was 2.3 compared to normal ONSD/ED, with an absolute risk difference of 43% (p = 0.04). Elevated ONSD/ED predicted long ICU-LOS with sensitivity 33%, specificity 93%, positive predictive value 75% and negative predictive value 68%. There was no significant difference in 90 day mortality. The 95% CIs were widely overlapping in the Kaplan Meier survival graph over 90 days (Table 2, Fig. 1). The difference in ICU-LOS remained significant through sensitivity analyses with exclusion of extreme values in ICU-LOS and cases of ICU-mortality (Table 3).

**Table 2:** Outcome measures reported and compared by normal or elevated ONSD/ED.

	Normal ONSD/ED, 43 patients	Elevated ONSD/ED, 11 patients	Absolute difference	p-value
ICU-LOS, mean (CI)	26 (95% CI 21 to 30)	38 (95% CI 26 to 50)	− 12 (95% CI − 23 to − 2)	0.03
Percentage ICU survivors with ICU-LOS ≥ 30 days	32%	75%	− 43%	0.04
90 days mortality, percentage	36%	35%	1%	1.00

*ICU-LOS* intensive care unit length of stay, *CI* 95% confidence interval, *ONSD/ED* optic nerve sheath diameter divided by eyeball diameter.

**Table 3:** Sensitivity analyses.

	Mean ICU-LOS (95% CI) and number of patients with normal ONSD/ED	Mean ICU-LOS (95% CI) and number of patients with elevated ONSD/ED	Difference (95% CI)	p-value
Excluding ICU-LOS > 50 days	22 (19 to 26) 38 patients	32 (22 to 42) 9 patients	− 10 (− 18 to 2)	0.03
Excluding ICU-LOS < 5 days	26 (22 to 31) 41 patients	38 (26 to 50) 11 patients	− 12 (− 22 to − 2)	0.03
Excluding ICU-LOS > 50 days or < 5 days	23 (20 to 26) 37 patients	32 (22 to 42) 9 patients	− 9 (− 17 to − 1)	0.04
Excluding cases of ICU mortality	24 (19 to 28) 36 patients	34 (23 to 45) 8 patients	− 10 (− 20 to 0)	0.04

*ICU-LOS* intensive care unit length of stay, *CI* confidence interval, *ONSD/ED* optic nerve sheath diameter divided by eyeball diameter.

There were no significant differences in age, sex, comorbidities or day of ONSD/ED measurement between patients with elevated ONSD/ED and normal ONSD/ED, respectively. Pregnancy, prior stroke, TBI or hydrocephalus/chronic intracranial hypertension were however too rare in this material to perform analyses of differences between the groups. There was one pregnant patient, three patients with previous stroke and one patient with previous TBI. ONSD/ED was normal in these five patients. There was no significant difference in the need for vasopressor or inotrope support, the occurrence of acute kidney injury, ventilator mode, ventilator settings, accumulated prone position time, PFI or pCO2 between the two groups (Table 4). There was no significant correlation between day of ONSD/ED measurement and measured ONSD/ED with a coefficient of 0.00 (95% CI − 0.00 to 0.00, p = 0.80).

**Table 4:** Comparison of baseline data by normal or elevated ONSD/ED.

Demographics	Normal ONSD/ED 43 patients	Elevated ONSD/ED 11 patients	p-value
Age, mean (CI)	63 (59 to 67)	66 (62 to 71)	0.12
Male, %	81%	73%	0.67
**Comorbidities**			
Cardiovascular disease, %	18%	18%	1.0
Asthma/COPD, %	14%	0	0.33
Diabetes, %	23%	36%	0.45
Obesity, %	37%	27%	0.73
Hypertension, %	35%	55%	0.31
**Treatments**			
Vasopressors at time of ONSD/ED measurement, %	67%	81%	0.47
Inotropes at time of ONSD/ED measurement, %	9%	18%	0.59
Ventilator mode at time of ONSD/ED measurement, pressure support, %	44.2%	54.5%	0.74
Ventilator mode at time of ONSD/ED measurement, pressure control, %	55.8%	45.5%	0.74
PEEP at time of ONSD/ED measurement, mean (CI)	11.2 (10.2 to 12.2)	11.5 (10.2 to 12.7)	0.73
Pressure support/pressure control at time of ONSD/ED measurement, mean (CI)	14.0 (12.7 to 15.4)	12.9 (10.0 t0 15.8)	0.45
Peak airway pressure at time of ONSD/ED measurement, mean (CI)	25.2 (23.7 to 26.8)	24.4 (20.9 to 27.8)	0.62
Accumulated time of prone positioning at time of ONSD/ED measurement, mean (CI)	38.2 (27.8 to 48.4)	37.1 (12.1 to 62.1)	0.93
**Other baseline data**			
PFI (ratio of pO2 in kPa to FiO2) at time of ONSD/ED measurement, mean (CI)	19 (17 to 21)	18 (14 to 22)	0.32
pCO2 (kPa) at time of ONSD/ED measurement, mean (CI)	6.7 (6.2 to 7.2)	6.0 (5.1 to 6.9)	0.08
Acute Kidney Injury (AKI) at time of ONSD/ED measurement, %	19%	18%	1.0
ICU day of ONSD/ED measurement, median (interquartile range)	6 (3 to 13)	8 (5 to 15)	0.74

*CI* confidence interval, *n* numbers, *COPD* chronic obstructive pulmonary disease, *ONSD/ED* optic nerve sheath diameter divided by eyeball diameter, *PFI* ratio of partial pressure of oxygen (kPa) in arterial blood divided by fraction of inspired oxygen, *pCO2* partial pressure of carbon dioxide (kPa) in arterial blood, AKI acute kidney injury, *ICU* intensive care unit.

## Discussion

This study shows that elevated ONSD/ED is common in severe Covid-19 and is associated with a significantly longer ICU-LOS in these patients. The association between ONSD/ED and ICU-LOS was not explained by baseline factors such as age, gender or co-morbidities, nor by timing of ONSD measurement during the ICU stay. It was also unrelated to ventilator modes or settings, accumulated prone positioning time, and the degree of hemodynamic, respiratory or renal failure at the time of ONSD/ED measurement.

Our findings closely match those of the only previous study estimating ICP noninvasively in patients with severe Covid-19 using ONSD sonography. In that study ONSD was measured in 49 patients and 10 of them (18.9%) were estimated to have an elevated ICP. These patients had a significantly longer ICU-LOS than the patients with an estimated normal ICP (45 days vs 36 days) but showed no significant differences in ICU- or hospital mortality^6^. We analyzed longer term outcome by comparing 90 day mortality between the groups. Another difference between that study and our study is that we corrected ONSD for ED.

Elevated ICP is not the only possible explanation to an elevated ONSD. Optic neuritis also leads to an increase in ONSD and has been reported in Covid-19^9,16^. It is however a rare complication to Covid-19 and therefore we deem it unlikely to cause the 20% occurrence of elevated ONSD that we see in this study. Prone positioning is a common treatment strategy in severe Covid-19. Prone positioning often leads to facial edema and in some cases elevated intra-ocular pressure^17^. It is unclear whether repeated prone positioning and accumulated prone positioning time affects ONSD. Prone positioning is daily performed in many patients with severe Covid-19 in our ICU setting and tends to increase with increasing severity of disease. If accumulated prone positioning time affects ONSD this would confound our findings. However, we detected no significant difference in accumulated prone positioning time between patients with normal and elevated ONSD/ED. Accumulated prone positioning time therefore cannot explain the association between ICU-LOS and ONSD/ED in our data.

ONSD has a well-established association with ICP^9,10^ and was not related to baseline factors or any of the potential confounders that were measured in our cohort. We therefore believe that the most likely interpretation of our results is that elevated ICP may be common and associated with adverse outcomes in Covid-19. There are several possible explanations to why elevated ICP would occur in severe Covid-19. High ventilator pressures and right ventricular failure are common in respiratory failure in general and specifically in severe Covid-19^18–21^. Both affect central venous pressure and thereby ICP^22–24^. Hypothetically, there could be a cumulative effect where sustained high ventilator pressures and sustained right ventricular failure over time would cause and exacerbate cerebral edema. High ventilator pressures are likely associated with longer ICU-LOS due to lung damage and is therefore a potential confounder in our study. There were however no significant differences in ventilator modes or ventilator pressures between patients with normal and elevated ONSD/ED at the time of ONSD/ED measurement in our data. Hypercapnia also may cause elevated ICP^25^. It is a common occurrence in late stage, severe Covid-19^20^ and is often permitted in respiratory failure to facilitate lung protective ventilation^19^. Again, no significant difference in pCO2 between patients with normal and elevated ONSD/ED was found in our data. We do not believe that either ventilator pressures or hypercapnia are driving factors behind the association between ONSD/ED and ICU-LOS. Systemic inflammation and neuroinflammation are other possible mechanisms of cerebral edema and therefore elevated ICP, alongside hypoxic lesions caused by hypoxemia, vascular complications and thromboembolic events. All of these can occur in severe Covid-19^1–3^ and are plausible explanations to our findings.

The suggestion that elevated ICP may be common in severe Covid-19, and is associated with adverse outcome, is clinically important. Firstly, because it provides a new perspective on the strategy of permissive hypercapnia mentioned above. Hypercapnia does not seem to be the driving factor behind our findings and pCO2 levels were overall moderate in our cohort. Higher levels of pCO2 will however further elevate ICP in patients with ICP instability^25^. Secondly, a practice has arisen, based on experiences from several ICUs, to sometimes position severe cases of Covid-19 flat or even in Trendelenburg position as a rescue maneuver. This is due to the paradoxical improvement in lung compliance sometimes witnessed in severe cases of Covid-19 in these positions, a phenomenon that recently was reviewed^26^. These positions as well as permissive hypercapnia will inevitably increase ICP^25,27^. No patients were in the Trendelenburg position when ONSD/ED was measured. If elevated ICP is a contributing factor to outcomes in severe Covid-19 this must be taken into consideration when discussing these mentioned treatment strategies. Likewise, treatment strategies regarding blood pressure targets, serum osmolality and dialysis doses in patients with acute kidney injury may need to be revised if elevated ICP truly is a factor in severe Covid-19. Further, the possibility to prognosticate ICU-LOS based on ONSD/ED may be clinically relevant. A reliable tool to predict length of ICU-LOS may inform such decisions as timing of tracheostomy and patient transfer. Prediction of ICU-LOS could provide valuable information for management decisions regarding allocation of resources.

There are limitations to this study, the most obvious being the small sample size. There may be a difference in 90 day mortality and associations between ICU-LOS and other parameters that this study was underpowered to detect. There were no statistically significant differences in comorbidities between the groups, however our small sample size may be underpowered to detect potentially true differences. Moreover, the small sample size may make results sensitive to outliers. Important to note though, our results were robust throughout sensitivity analyses, as previously described. The convenience sample strategy that was necessary to perform this study during an ongoing pandemic made it vulnerable to selection bias. Also, this strategy led to ICP estimation being performed at different times during the patients’ ICU stay. This might have affected the results if ONSD changed through the course of the disease. But since the day of ONSD/ED measurement was not correlated to ONSD/ED, we do not believe this to interfere with results. Outcome measures pose another set of limitations in this study. ICU-LOS is prone to confounding by ICU-mortality but our findings were robust throughout sensitivity analysis excluding cases of ICU mortality. Neither ICU-LOS nor survival yield information regarding long term quality of life or neurological function. This may be one of the greatest limitations of this study. Finally, it should be stressed that ONSD sonography does not yield precise values of ICP. It is an ICP surrogate and false positives are to be expected. Also, there is no consensus regarding ONSD/ED cut-off to identify elevated ICP. Our cut-off at 0.295 mm is based on unpublished data currently undergoing peer review.

Given that ONSD/ED showed no association with baseline factors or any of the potential confounders we measured, we still believe that elevated ICP is the most likely explanation for elevated ONSD/ED in this cohort.

## Conclusions

We conclude that elevated ONSD/ED is common in severe Covid-19, is associated with adverse outcome and can predict ICU-LOS ≥ 30 days. These results are in line with results from the only similar previous study. Having analyzed several important potential confounders, we believe that the remaining and most likely explanation for this is that elevated ICP occurs and correlates with, or contributes to, morbidity in severe Covid-19. This would have clinical implications and should therefore prompt further studies into possible mechanisms and treatment strategies. We recommend that ongoing or future studies of ICP in severe Covid-19 should be performed with larger cohorts, recording exact data on patient positioning and including more patient-centered outcome measures.

## Data Availability

The datasets generated and/or analysed during the current study are not publicly available due to constraints in the ethical permission granted by the Swedish Ethical Review Authority but are available from the corresponding author on reasonable request. The ethical approval for this study allows publication of aggregated data only.
